# Rational use of drugs to alleviate adverse outcomes caused by COVID-19 quarantine in women with intrahepatic cholestasis of pregnancy

**DOI:** 10.3389/fmed.2023.1122873

**Published:** 2023-08-07

**Authors:** Qin-Yu Cai, Xia Li, Yin Yang, Xin Luo, Shu-Juan Luo, Jing Xiong, Zong-Yan He, Yuan Chen, Yi-Wei Mou, Ji-Yuan Hu, Shu Yang, Xia Lan, Tai-Hang Liu

**Affiliations:** ^1^Department of Bioinformatics, The School of Basic Medicine, Chongqing Medical University, Chongqing, China; ^2^The Joint International Research Laboratory of Reproduction and Development, Ministry of Education, Chongqing, China; ^3^Department of Infection Controlling Section, Women and Children's Hospital of Chongqing Medical University, Chongqing, China; ^4^Department of Obstetrics, The First Affiliated Hospital of Chongqing Medical University, Chongqing, China; ^5^Department of Obstetrics and Gynecology, Women and Children's Hospital of Chongqing Medical University, Chongqing, China

**Keywords:** ICP, COVID-19, home quarantine, ursodeoxycholic acid, pregnancy outcome, public health

## Abstract

**Purpose:**

This study aimed to investigate the impacts of home quarantine on pregnancy outcomes of women with intrahepatic cholestasis of pregnancy (ICP) during the COVID-19 outbreak and whether the rational use of drugs will change these impacts.

**Methods:**

This multi-center study was conducted to compare the pregnancy outcomes in women with ICP between the home quarantine group and the non-home quarantine group in southwest China. Propensity score matching was performed to confirm the pregnancy outcomes of the medication group and the non-medication group in women with ICP during the epidemic period.

**Results:**

A total of 3,161 women with ICP were enrolled in this study, including 816 in the home quarantine group and 2,345 in the non-home quarantine group. Women with ICP in the home quarantine group had worse pregnancy outcomes, such as a growing risk of gestational diabetes mellitus A1, fetal growth restriction, pre-eclampsia, preterm delivery, and even stillbirth. Drug therapy could alleviate some adverse pregnancy outcomes caused by home quarantine, including pre-eclampsia, preterm delivery, and meconium-stained amniotic fluid.

**Conclusion:**

COVID-19 quarantine would increase the incidence of ICP and lead to adverse pregnancy outcomes in women with ICP. The rational use of drugs reduced some obstetrical complications and improved partial pregnancy outcomes. Our findings suggested that the government and hospitals should enhance their management and life guidance for women with ICP and speed up developing home quarantine guidelines.

## Introduction

In 2019, a novel coronavirus disease, COVID-19 (caused by the severe acute respiratory syndrome coronavirus 2, SARS-CoV-2), emerged in Wuhan, China. Since then, the virus and its variants have continued to spread rapidly across the globe, infecting hundreds of millions of people and causing millions of deaths. Worse still, COVID-19 infection has been linked to several adverse health outcomes, including neurological, cardiovascular, and pulmonary sequelae (1). As a result, governments worldwide have implemented various degrees of lockdown measures to curb the spread of the virus. However, the lockdown has proved to be a double-edged sword, significantly altering people's daily lives and affecting their work, physical activities, sleep, and dietary habits (2, 3). These changes have been associated with numerous adverse effects, including psychological disorders among adolescents, domestic violence, exacerbation of chronic diseases, and adverse pregnancy outcomes (4–6).

Intrahepatic cholestasis of pregnancy (ICP) is a liver disease that occurs exclusively during pregnancy and is characterized by symptoms such as pruritus, jaundice, and elevated bile acids. While the symptoms and biochemical abnormalities typically disappear soon after delivery, there is a high probability of relapse during subsequent pregnancies or with hormonal contraception. ICP increases the risk of maternal hepatobiliary diseases and can lead to adverse perinatal outcomes, including fetal ischemia and hypoxia, spontaneous preterm delivery, meconium-stained amniotic fluid, neonatal respiratory distress, and stillbirth (7). The incidence of ICP varies widely depending on the geographical location and ethnicity, ranging from 0.3% to 15% (8). Worldwide, the incidence of ICP is higher in South America and northern Europe, and it is most common in the Yangtze River Basin in China, including Chongqing, Sichuan, and the Yangtze River Delta. Currently, the etiology of ICP has not been fully elucidated, but it is believed to be related to immune abnormalities, genetics, environmental factors, viral hepatitis, and hormonal changes (9). Research has demonstrated that reducing exercise does not promote weight, blood sugar, or bile acid control. Consuming an unhealthy diet, such as one that is high in carbohydrates, with less nutrients, and low in fiber, can increase cholesterol levels and result in elevated bile acid levels (10). With the current trend of home quarantine, individuals may develop unhealthy eating and exercise habits, which can lead to elevated bile acid levels. Bile acid is commonly used as a biomarker for diagnosing ICP in clinical practice, as indicated by various national and regional guidelines, including those in China (11–13). Studies suggested that bile acid levels were associated with an increased risk as well as a predictor of adverse pregnancy outcomes such as stillbirth, fetal growth restriction (FGR), and meconium-stained amniotic fluid (14). Additionally, the home quarantine has contributed to a rise in maternal depression or mental health issues, which have been linked to short-term and long-term adverse outcomes for pregnant women and newborns (15, 16). Furthermore, the home quarantine has also had detrimental effects on antenatal care, leading to missed medical treatments, delayed diagnoses, and reduced doctor visits, ultimately contributing to adverse pregnancy and birth outcomes among women (17). Consequently, we suspect that home quarantine may increase the incidence of ICP and lead to more adverse pregnancy outcomes in pregnant women with ICP. However, there is currently no definitive evidence to support this hypothesis.

Here, a multi-center retrospective cohort study was conducted to evaluate the effects of home quarantine on pregnancy outcomes among women with ICP. The study included pregnant women with ICP who underwent strict home quarantine between 24 January 2020 and 20 April 2020 in Chongqing, China. These women were classified as the home quarantine group. The non-home quarantine group consisted of pregnant women with ICP without epidemic lockdown during the same period in 2018, 2019, and 2021. The results of this study are anticipated to provide valuable guidance for antenatal care and clinical decision-making for women with ICP during public health emergencies.

## Materials and methods

### Ethics approval

This study has been approved by the ethics committee of *Chongqing Medical University* (ID: 20220627). During the process of data collection and analysis, all personally identifiable information of the pregnant women was deleted to protect their privacy.

### Study design

**Figure 1:**
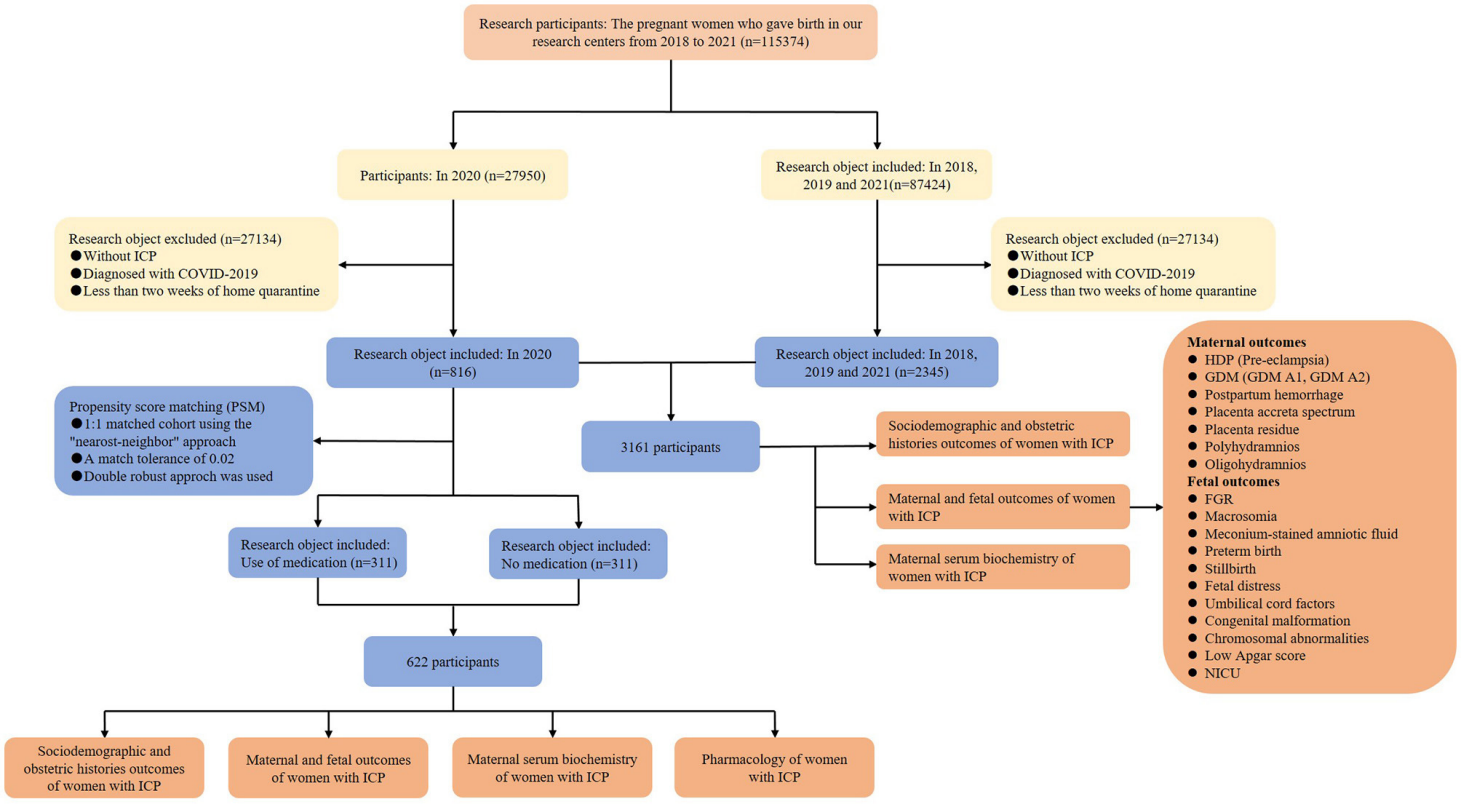
The flowchart of the study.

This multicenter retrospective study was conducted at two Grade III and Grade A hospitals in Chongqing, China, including the First Affiliated Hospital of Chongqing Medical University and the Women and Children's Hospital of Chongqing Medical University (Figure 1). The total number of newborns in the two hospitals is over 10,000 and 15,000 per year, respectively, making them the two largest maternal hospitals in Chongqing. The electronic medical records of all pregnant women, including their sociodemographic and obstetric histories, as well as clinical information, were systematically preserved in both hospitals. This study aimed to unveil the impacts of home quarantine exposure on pregnancy outcomes in women with ICP and to determine whether medication affects pregnancy outcomes. The study cohort included pregnant women with ICP who had undergone at least 2 weeks of home quarantine during the COVID-19 epidemic as well as women without home quarantine during the same period in 2018, 2019, and 2021. The inclusion criteria for home quarantine were selected based on similar studies, requiring at least 2 weeks of quarantine (18).

We inferred that the pregnant women who delivered from 7 February 2020 to 31 December 2020 experienced a mandatory home quarantine of at least 2 weeks as per the strict guidelines imposed in Chongqing, China, during the period of 24 January 2020 to 20 April 2020. Subsequently, the complete clinical data of pregnant women from 7 February 2020 to 31 December 2020 were collected and categorized as the home quarantine group. Additionally, the clinical data of pregnant women during the same period in 2018, 2019, and 2021 (birth years) were also collected and jointly grouped as the non-home quarantine group (control group) due to the fact that 2020 is the COVID-19 quarantine year that was not included. ICP diagnosis was strictly based on the international diagnostic clinical criteria: mild ICP was diagnosed with the complaint of pruritus and a fasting serum bile acid concentration greater than 10 μmol/L, and women were considered to have severe ICP with the fasting serum bile acid level >40 μmol/L (11, 19). Exclusion criteria were women without ICP or diagnosed with the COVID-19 virus.

### Data collection

All demographic and clinical data, including maternal information and outcomes as well as neonatal outcomes, were obtained from the electronic medical records of two hospitals. Two data collectors extracted medical records simultaneously, and any discrepancies in their descriptions were reviewed and reconciled to ensure accurate data extraction. Furthermore, all data collectors did not know the main purpose and hypothesis of the study.

### Definitions

The effects of home quarantine on maternal and fetal adverse outcomes were the critical content of this study. Maternal outcomes include the mode of delivery, gestational diabetes mellitus A1 (GDM A1, treated with dietary therapy), gestational diabetes mellitus A2 (GDM A2, needed pharmacologic treatment), gestational hypertension, pre-eclampsia, postpartum hemorrhage (defined as >1000 ml), placenta accreta spectrum, placenta residue, polyhydramnios (defined as an amniotic fluid index (AFV) of >2,000 ml), and oligohydramnios (defined as an AFV that is < 200 or 500 ml) (20, 21). GDM was defined as the following criterion: after 8–10 h of overnight fasting, all the pregnant women (24–28 gestational weeks) were given a 75 g oral glucose tolerance test; the diagnosis was made when any of the following criteria were met according to the recommendations of the International Association of the Diabetes and Pregnancy Study Groups Consensus Panel (IADPSG/WHO), including fasting glucose ≥5.1 mmol/L, 1 h glucose ≥10.0 mmol/L, or 2 h glucose ≥8.5 mmol/L (22). Diagnosis of gestational hypertension (HDP), according to the International Association for the Study of Hypertension in Pregnancy, is defined as systolic blood pressure ≥140 mm Hg and/or diastolic blood pressure ≥90 mm Hg at least two times apart, occurring after 20 weeks of gestation without significant proteinuria. Then, pre-eclampsia was diagnosed by hypertension accompanied by proteinuria ≥300 mg in 24 h or at least two readings of “++” on the dipstick in the test of midstream or catheter urine samples within 24 h (23).

Fetal outcomes include small for gestational age (defined as birthweight < 10th percentile), FGR (fetal weight below the third percentile), macrosomia (birthweight >4,000 g), meconium-stained amniotic fluid, preterm birth (defined as < 37 weeks of gestation), fetal distress, umbilical cord factors (umbilical cord around the neck or umbilical cord knot), congenital malformation, chromosomal abnormalities, low Apgar score (< 7 after 5 min), admissions to medium care unit and neonatal intensive care unit, and stillbirth (24 weeks of gestation to 7 days after delivery). All outcome measures are based on previous large-scale studies on comparative programs and national guidelines (24, 25).

### Statistical analyses

The descriptive analysis of the collected data was conducted using statistical analysis to describe categorical and continuous variables. The mean ± SD was used to manifest the continuous variables with a normal distribution, including gestational weight gain and maternal age. Then, the continuous variables that do not obey normal distribution were manifested as median plus 25–75 interquartile range (IQR), such as gestational age, serum bile acids, total bilirubin, and maternal serum biochemistry. Additionally, frequency and percentages were shown to describe categorical variables, including gravidity, parity, body mass index, and other sociodemographic and obstetric histories or maternal and fetal outcomes of women with ICP. The difference analysis of continuous variables with a normal distribution was conducted using an unpaired Student's *t*-test. Otherwise, the equivalent nonparametric test, the Kruskal–Wallis H-test, was used to analyze the differences between the home quarantine and the control groups. Chi-square or Fisher's exact tests were used to compare the categorical variables. Moreover, body mass index was categorized into categorical variables rather than continuous ones. Afterward, univariate logistic regression was used to analyze the maternal and fetal outcomes of women with ICP in the home quarantine and the non-home quarantine groups. All results show an odds ratio (OR) and 95% confidence interval (CI). Then, all factors with *P* < 0.10 were considered possible confounding factors and evaluated by the backward elimination strategy in multivariate logical regression analysis, including gestational weight gain, birth plurality, use of medication, and skin itch. These data were presented in the form of adjusted odds ratios (AOR) and 95% CIs.

Owing to the significant interaction between women with ICP and medication use in the regression model for the total cohort (*P* < 0.001), subgroup analyses were further conducted in the home quarantine group, including medication and no medication subgroups. To reduce any known confusion caused by selection bias, propensity score matching (PSM) was performed to balance the baseline characteristics of the two groups and reduce potential confounding factors. PSM was conducted to get a 1:1 matched cohort after using the “nearest-neighbor” approach without replacement, and a match tolerance of 0.02 was used. The propensity score was defined as the predicted probability of the patient using medication given the sociodemographic and obstetric histories of pregnant women, maternal serum biochemistry, and comorbidities. Furthermore, the covariates used to calculate the propensity score included body mass index, birth plurality, history of ICP, assisted reproductive technology, the severity of ICP, and skin itch. The factors used to calculate the propensity score included maternal age, gestational weight gain, and gestational age. To obtain a balanced distribution of all the covariates in the PSM cohort, if any baseline features did not satisfy the balanced distribution, a double robust method was performed to eliminate any residual confounding deviations after PSM (26). All outcomes of the PSM cohorts were based on statistical analysis, the same as the above methods used in the total cohort. A *p* < 0.05 was considered significant. Because the missing value in the variables used in the statistical analysis was not more than 5%, no extrapolation was made on the missing data in the analysis. All data refer to the number of pregnant women enrolled in our cohort study, and all analyses were conducted by Statistical Package for the Social Sciences 26.0 software (SPSS 26.0, IBM Corporation, Chicago, USA).

## Results

### Sociodemographic and obstetric histories of pregnant women with ICP in home and non-home quarantine pregnancies

After screening the electronic medical records of the pregnant women in the two hospitals, the data revealed that a total of 115,374 pregnant women gave birth from 2018 to 2021; 27,950 in 2020; and 87,424 in the remaining 3 years. Among them, 27,134 pregnant women who gave birth in 2020 were excluded due to being without ICP, being diagnosed with COVID-19, or having < 2 weeks of home quarantine, and 85,071 in 2018, 2019, and 2021 were excluded according to the same criteria. After taking the inclusion and exclusion criteria into account, 816 participants with ICP were classified into the home quarantine group, while the remaining 2,345 women with ICP were included in the control group. Thus, the cohort comprised a total of 3,161 participants (Figure 1). The analysis of the incidence of ICP in two groups showed that the incidence of ICP rate in the home quarantine group was 2.92%, which was significantly higher than that in the non-home quarantine group (2.68%; *P* = 0.031, Supplementary Figure S1). Comparisons of the sociodemographic and obstetric histories of pregnant women with ICP between the home quarantine group and the non-home quarantine group are shown in Supplementary Table S1. Women with ICP in the home quarantine group were more likely to gain gestational weight than in the non-home quarantine group (*P* = 0.035). Moreover, the proportion of twins in the home quarantine group (19.2%, 157 women) was higher than in the non-home quarantine group (16.0%, 375 women), which was shown in further analysis (*P* = 0.033). Women with ICP in the home quarantine group have fewer symptoms of skin itch (30.9%, 252 women vs. 38.9%, 912 women, *P* < 0.001). Although more than one-third of women received medication, the proportion of pregnant women who used medication to treat ICP in the home quarantine group was lower than that in the control group (39.5%, 322 women vs. 47.6%, 1,166 women, *P* < 0.001). In addition, there were no significant differences in gestational age, maternal age, gravidity, parity, abortion, pre-gestation body mass index (BMI), cigarette smoking, alcohol consumption, the severity of ICP, history of hepatobiliary disease, history of ICP, or assisted reproductive technology between the two groups. These suggested that the baseline characteristics of the two groups were similar. The characteristics with significant differences in sociodemographic and obstetric histories were identified as potential confounding factors. The multiple regression model was used for post-adjustment analysis in the subsequent analysis.

### Maternal and fetal outcomes of women with ICP in home and non-home quarantine pregnancies

Then, the impacts of home quarantine on obstetric outcomes were analyzed, and the details of the results are manifested in Table 1. The data showed that women with ICP in the home quarantine group had a growing risk of GDM A1 (AOR 1.34, 95% CI 1.09–1.67, *P* = 0.007) and pre-eclampsia (AOR 1.63, 95% CI 1.02–2.60, *P* = 0.041); moreover, the significant difference remained after following multiple logistic regression to adjust potential confounding factors, including birth plurality, gestational weight gain, skin itch, and use of medication. Additionally, women with ICP in the home quarantine group also had an increased risk of postpartum hemorrhage (AOR 1.72, 95% CI 1.02–2.89, *P* = 0.043) and placenta accreta spectrum (AOR 1.42, 95% CI 1.06–1.91, *P* = 0.019). However, there was no significant difference in GDM A2, pre-eclampsia, placenta residue, or oligohydramnios.

**Table 1:** Maternal and fetal outcomes of women with ICP in the home quarantine and non-home quarantine groups.

	**Home quarantine**	**Non-home quarantine**	**Crude**		**Adjusted**	
**Outcomes**	**n (%)**	**n (%)**	**OR (95% CI)**	***P***	**OR (95% CI)**	***P*** ^§^
**Maternal outcomes**						
**Mode of delivery**			1.00 (0.84–1.00)	0.996	1.16 (0.97–1.39)	0.115
Cesarean delivery	568 (69.7%)	1,634 (69.7%)				
Vaginal delivery	248 (30.3%)	711 (30.3%)				
Forceps assisted	10 (1.2%)	48 (2.0%)	0.59 (0.30–1.18)	0.136	0.60 (0.30–1.20)	0.147
Gestational diabetes mellitus A1^**†**^	157 (19.2%)	350 (14.9%)	1.36 (1.10–1.67)	0.004^*^	1.34 (1.09–1.67)	0.007^*^
Gestational diabetes mellitus A2	12 (1.5%)	51 (2.2%)	0.67 (0.36–1.26)	0.216	0.61 (0.31–1.20)	0.153
Gestational hypertension	22 (2.7%)	55 (2.3%)	1.03 (0.73–1.45)	0.859	1.06 (0.75–1.51)	0.737
Pre-eclampsia^**†**^	57 (7.0%)	121 (5.2 %)	1.59 (1.01–2.50)	0.045^*^	1.63 (1.02–2.60)	0.041^*^
Postpartum hemorrhage^**†**^	24 (2.9%)	40 (1.7%)	1.74 (1.04–2.90)	0.035^*^	1.72 (1.02–2.89)	0.043^*^
Placenta accreta spectrum^**†**^	75 (9.2%)	163 (7.0%)	1.35 (1.01–1.60)	0.038^*^	1.42 (1.06–1.91)	0.019^*^
Placenta residue	4 (0.5%)	16 (0.7%)	0.72 (0.24–2.15)	0.717	0.69 (0.23–2.12)	0.522
Polyhydramnios^**†**^	36 (4.4%)	64 (2.7%)	1.64 (1.08–2.49)	0.020^*^	1.83 (1.19–2.81)	0.006^**^
Oligohydramnios	50 (6.1%)	171 (7.3%)	0.83 (0.60–1.15)	0.261	0.75 (0.54–1.04)	0.082
**Fetal outcomes**						
Low birth weight^**†**^	135 (16.5%)	361 (15.4%)	1.09 (0.88–1.35)	0.442	1.40 (1.10–1.78)	0.007^**^
Fetal growth restriction^**†**^	30 (3.7%)	50 (2.1%)	1.74 (1.10–2.76)	0.018^*^	1.93 (1.20–3.10)	0.006^**^
Macrosomia (birthweight >4,000 g)	21 (2.6%)	63 (2.7%)	0.84 (0.95–1.57)	0.837	0.88 (0.53–1.47)	0.623
Preterm delivery^**†**^	146 (17.9%)	395 (16.9%)	1.07 (0.87–1.32)	0.510	1.40 (1.11–1.76)	0.004^**^
Stillbirth^**†**^	5 (0.6%)	4 (0.2%)	3.45 (1.05–6.30)	0.041^*^	5.15 (1.98–10.06)	0.005^**^
Fetal distress	66 (8.2%)	178 (7.7%)	1.06 (0.79–1.42)	0.696	0.96 (0.71–1.30)	0.811
Meconium-stained amniotic fluid^**†**^	152 (18.6%)	341 (14.6%)	1.3491.10–1.65)	0.007^**^	1.25 (1.10–1.55)	0.044^*^
Umbilical cord factors^**†**^	240 (29.4%)	596 (25.5%)	1.16 (0.97–1.39)	0.113	1.22 (1.02–1.45)	0.029^*^
Fetus with congenital malformation^**†**^	12 (1.5%)	29 (1.2%)	1.19 (0.60–2.34)	0.615	1.16 (0.58–2.31)	0.591
Fetal chromosomal abnormalities	2 (0.2%)	15 (0.6%)	0.19 (0.03–1.44)	0.108	0.18 (0.02–1.35)	0.094
1 min Apgar score < 7	5 (0.6%)	6 (0.3%)	2.40 (0.73–7.89)	0.149	3.32 (0.96–11.421)	0.057
5 min Apgar < 7	2 (0.2%)	2 (0.1%)	2.88 (0.41–20.47)	0.291	4.78 (0.63–28.51)	0.180
Intensive care unit admission^**†**^	120 (14.7%)	266 (11.4%)	1.34 (1.06–1.69)	0.014^*^	1.66 (1.30–2.13)	< 0.001^***^

Home quarantine was used as the reference group.

^†^There was a statistically significant difference between the home quarantine group and the non-home quarantine group.

^§^Adjust: gestational weight gain, birth plurality, using of medication, and skin itch.

^*^P < 0.05, ^**^P < 0.01, ^***^P < 0.001.

Through further analysis of fetal outcomes, the data showed that the fetuses in the home quarantine group had an increased risk of low birth weight (AOR 1.40, 95% CI 1.10–1.78, *P* = 0.007), FGR (AOR 1.93, 95% CI 1.12–3.10, *P* = 0.006), and preterm delivery (AOR 1.40, 95% CI 1.11–1.76, *P* = 0.004). Furthermore, the fetuses in the home quarantine group were more prone to happen with meconium-stained amniotic fluid (AOR 1.25, 95% CI 1.10–1.55, *P* = 0.044), a umbilical cord factors (including the umbilical cord around the neck or umbilical cord knot; AOR 1.22, 95% CI 1.02–1.45, *P* = 0.029), and a low Apgar score (< 7) at 1 min (AOR 4.05, 95% CI 1.24–13.21, *P* = 0.020). Therefore, further analysis showed that fetuses in the home quarantine group had a higher risk of intensive care unit admission (AOR 1.66, 95% CI 1.30–2.33, *P* < 0.001). More importantly, there were five stillbirths (0.6%) in the home quarantine group, which were significantly higher than the four stillbirths (0.2%) in the non-home quarantine group (AOR 5.15, 95% CI 1.98–10.06, *P* = 0.005).

The analysis of the maternal serum biochemistry of women with ICP in the home quarantine group and the non-home quarantine group is shown in Supplementary Table S2. The biochemistry markers of liver function showed that the maternal serum bile acids of women with ICP (*P* = 0.049) in the home quarantine group were higher than those in the non-home quarantine group at the delivery stage. The other indexes, including alanine transaminase (ALT), aspartate aminotransferase (AST), and total bilirubin, showed no significant difference at the diagnosis and delivery stages.

### Impacts of medication on the maternal and fetal outcomes of women with ICP in the home quarantine group

We further explored whether rational drug use to treat ICP could improve adverse pregnancy outcomes during home quarantine. Most of the drugs were recommended for the treatment of maternal symptoms of ICP with high evidence grade or common use in clinical practice to decrease the bile acid level in China, mainly including ursodeoxycholic acid, polyene phosphatidylcholine, S-adenosyl methionine, reduced glutathione tablets, and compound glycyrrhizin. Moreover, all the drugs used were prescribed by doctors based on the detailed clinical symptoms among women with ICP. After making PSM in the home quarantine group, 311 cases were well matched between the two groups or 622 cases in total, and the generated results balanced sociodemographic and obstetric histories (Supplementary Table S3). First, the sociodemographic and obstetric histories of pregnant women with home quarantine were compared between the medication and non-medication groups. The data showed that all baseline characteristics showed no significant difference between the two groups except for skin itch (*P* = 0.001).

The details of the obstetric outcomes of pregnant women with home quarantine in the medication group and the non-medication group are shown in Table 2. The pregnant women in the non-medication group had an increased risk of pre-eclampsia (AOR 3.00, 95% CI 1.31–6.87, *P* = 0.009) and polyhydramnios (AOR 4.10, 95% CI 1.21–13.93, *P* = 0.024), and the significant difference remained after adjusting the only confounding factors of skin pruritus by the multivariate logistic regression analysis. The fetuses in the non-medication group were more likely to have preterm delivery (AOR 1.69, 95% CI 1.10–2.59, *P* = 0.017), abnormal umbilical cord factors (AOR 1.82, 95% CI 1.26–2.63, *P* < 0.001), and meconium-stained amniotic fluid (AOR 1.49, 95% CI 1.05–2.34, *P* = 0.048). Moreover, the fetuses had a higher risk of intensive care unit admission (AOR 1.71, 95% CI 1.07–2.74, *P* = 0.025).

**Table 2:** Maternal and fetal outcomes of women with ICP who used medication or without medication in the home quarantine group.

	**Medication**	**Non-medication**	**Crude**		**Adjusted**	
**Outcomes**	***n*** **(%)**	***n*** **(%)**	**OR (95% CI)**	***P***	**OR (95% CI)**	***P*** ^§^
**Maternal outcomes**						
Mode of delivery			0.77 (0.55–1.09)	0.138	0.79 (0.55–1.14)	0.205
Cesarean delivery	226 (72.7%)	209 (67.2%)				
Vaginal delivery	85 (27.3%)	102 (32.8%)				
Forceps assisted	5 (1.6%)	2 (0.6%)	0.40 (0.008–2.06)	0.271	0.32 (0.06–1.73)	0.187
Gestational diabetes mellitus A1	56 (18.0%)	53 (17.0%)	0.94 (0.62–1.42)	0.752	0.97 (0.03–1.50)	0.897
Gestational diabetes mellitus A2	4 (1.3%)	4 (1.3%)	1.00 (0.25–4.04)	1.000	0.83 (0.20–3.49)	0.801
Gestational hypertension	8 (2.6%)	11 (3.5%)	1.39 (0.55–3.50)	0.486	1.36 (0.52–3.60)	0.534
Pre-eclampsia^**†**^	8 (2.6%)	27 (8.7%)	3.60 (1.61–8.06)	0.002^**^	3.00 (1.31–6.87)	0.009^**^
Postpartum hemorrhage	5 (1.6%)	10 (3.2%)	2.03 (0.69–6.02)	0.200	2.04 (0.68–6.07)	0.222
Placenta accreta spectrum	23 (7.4%)	34 (10.9%)	0.65 (0.37–1.13)	0.129	0.73 (0.41–1.30)	0.279
Placenta residue	2 (0.6%)	2 (0.6%)	1.00 (0.14–7.14)	1.000	0.70 (0.10–5.00)	0.721
Polyhydramnios^**†**^	4 (1.3%)	12 (3.9%)	3.08 (0.98–9.66)	0.054	4.10 (1.21–13.93)	0.024^*^
Oligohydramnios	16 (5.1%)	27 (8.7%)	1.75 (0.93–3.32%)	0.085	1.61 (0.83–3.13)	0.164
**Fetal outcomes**						
Low birth weight	48 (15.4%)	56 (18.0%)	1.20 (0.79–1.84)	0.390	1.40 (0.89–2.21)	0.142
Fetal growth restriction	7 (2.3%)	16 (5.1%)	2.36 (0.96–5.81)	0.063	3.25 (1.22–4.71)	0.347
Macrosomia (birthweight >4,000 g)	7 (2.3%)	10 (3.2%)	1.44 (0.54–3.84)	0.463	1.65 (0.58–4.71)	0.347
Preterm delivery^**†**^	54 (17.4%)	70 (22.5%)	1.38 (0.93–2.05)	0.109	1.69 (1.10–2.59)	0.017^*^
Stillbirth	1 (0.3%)	2 (0.6%)	2.01 (0.18–10.24)	0.570	2.59 (0.20–14.12)	0.470
Fetal distress	23 (7.4%)	28 (9.0%)	0.81 (0.45–1.44)	0.466	0.72 (0.39–1.32)	0.286
Meconium-stained amniotic fluid^**†**^	42 (13.5%)	60 (19.3%)	1.53 (0.99–2.35)	0.052	1.49 (1.05–2.34)	0.048^*^
Umbilical cord factors^**†**^	77 (24.8%)	112 (36.0%)	1.71 (1.21–2.42)	0.002^*^	1.82 (1.26–2.63)	0.001^**^
Fetus with congenital malformation	5 (1.6%)	2 (0.6%)	0.40 (0.08–2.06)	0.271	0.32 (0.06–1.73)	0.187
Fetal chromosomal abnormalities	1 (0.3%)	1 (0.3%)	1.00 (0.06–16.06)	1.000	1.56 (0.08–31.32)	0.773
1 min Apgar score < 7	2 (0.6%)	3 (1.0%)	2.01 (0.37–11.07)	0.421	1.90 (0.32–11.31)	0.482
5 min Apgar < 7	1 (0.3%)	1 (0.3%)	1.00 (0.62–16.06)	1.00	0.64 (0.03–12.94)	0.773
Intensive care unit admission^**†**^	39 (12.5%)	57 (18.3%)	1.57 (1.01–2.43)	0.047^*^	1.71 (1.07–2.74)	0.025^*^

The medication group was used as the reference group.

^†^There was a statistically significant difference between the medication group and non-medication group.

^§^Adjust: skin itch.

^*^P < 0.05, ^**^P < 0.01.

Subsequently, the maternal serum biochemistry of pregnant women with home quarantine was analyzed between the medication and non-medication groups (Supplementary Table S4). The results demonstrated that the levels of serum bile acids (*P* = 0.042), ALT (*P* = 0.025), and total bilirubin (*P* = 0.003) in the medication group were lower than that in the non-medication group at the delivery stage. The other serum bile acids and biochemistry indexes, including ALT, AST, and total bilirubin, showed no significant difference at the diagnosis and delivery stages.

### Pharmacology of pregnant women with ICP in the home quarantine group

In the cohort of the medication group, pregnant women with ICP used one or more drugs to treat ICP according to their actual clinical conditions. Further details of pharmacological management are shown in Table 3. Results showed that ursodeoxycholic acid (UDCA) had been taken by 82.6% of women (266 women) to treat ICP, which was recommended as the first-line agent for the treatment of maternal symptoms of ICP (GRADE 1A). The doses used ranged from 150 mg to 2 g per day, according to the specific situation of the women (25). As the second most frequently used drug, polyene phosphatidylcholine was used by 52.8% of women (177 women). As an alternative drug, S-adenosyl methionine was considered for women with ICP who could not take UDCA or who had continued symptoms on the maximum dosage of UDCA, and it was given to 39.4% of women (127 women). Reduced glutathione tablets (11.2%, 36 women) and compound glycyrrhizin (A traditional Chinese medicine that can reduce bile acid; 4.7%, 15 women) were also used to relieve the symptoms of ICP in the clinic. In addition, dexamethasone (21.1%, 68 women) and magnesium sulfate (6.2%, 20 women) were also used to improve fetal outcomes when necessary.

**Table 3:** Pharmacology of women with ICP in home quarantine.

**Drug therapy**	***N* = 322 (%)**
Ursodeoxycholic acid	266 (82.6%)
Polyene phosphatidylcholine	170 (52.8%)
S-adenosyl methionine	127 (39.4%)
Reduced glutathione tablets	36 (11.2%)
Compound glycyrrhizin	15 (4.7%)
Dexamethasone	68 (21.1%)
Magnesium sulfate	20 (6.2%)

All results were given as frequency and percentages.

### Perinatal mortality

Further analysis of stillbirths showed five stillbirths were found in the home quarantine group and four in the control group, with an incidence of 0.6% and 0.2%, respectively. All the characteristics of stillbirth cases are shown in Table 4. One woman with ICP was pregnant with twins in the home quarantine group, while the control group was all singletons. The median maternal age in the home quarantine group was 29.00 (IQR 26.75–31.00) and 32.00 (IQR 26.00–35.50) in the control group, which revealed the control group had advanced maternal age. Then, the median gestational age at delivery in the home quarantine population was 34.00 (IQR 32.72–37.93) and the control group was 32.72 (IQR 30.40–36.86) for stillbirth cases. These results showed women with ICP in the home isolation group were closer to the normal delivery time when stillbirth happened. Four-fifths of ICP cases (four patients) had coexistent pregnancy complications, including one case with pre-eclampsia, two with GDM A1, and one with GDM A2 in the home quarantine group. The maternal serum bile acids of women with ICP in the home quarantine group (median 49.8; IQR 35.8–81.5) were higher than those in the control group (median 43.0; IQR 29.6 to 68.4, *P* = 0.037) in the delivery stage. There were three neonatal deaths among the women with ICP after delivery with meconium-stained amniotic fluid in the home quarantine group and only one neonatal death in the control group. Three cases in the home quarantine group and three in the control group had used drugs to treat ICP, and all of them had used UDCA or polyene phosphatidylcholine. Additionally, all the women with ICP in the home quarantine group who used medication showed clinical symptoms of skin itch when they visited the doctor. The control group is basically the same; only one patient had no skin itch.

**Table 4:** Characteristics of stillbirth cases.

**Characteristics**	**Home quarantine (*n* = 5)**	**Non-home quarantine (*n* = 4)**
Incidence	0.6%	0.2%
Twins	1 (20.0%)	0 (0.0%)
Maternal age	29.00 (26.75–31.00)	32.00 (26.00–35.50)
Gestational age at delivery	34.00 (32.72–37.93)	32.72 (30.40–36.86)
Serum bile acids	49.8 (35.8–81.5)	43.0 (29.6–68.4)
Pre-eclampsia	1 (20.0%)	1 (25.0%)
Gestational diabetes A1	2 (40.0%)	2 (50.0%)
Gestational diabetes A2	1 (20.0%)	0 (0.0%)
Meconium-stained amniotic fluid	3 (60.0%)	1 (25.0%)
Use of medication	3 (60.0%)	3 (75.0%)
Skin itch	5 (100.0%)	3 (75.0%)

## Discussion

The COVID-19 pandemic continues to pose myriad challenges to global public health. While stay-at-home orders of varying degrees and forms have proven effective in preventing the virus from spreading more widely and protecting the population, sudden home quarantine can further disadvantage vulnerable groups, especially pregnant women. Several studies have reported that home quarantine can have serious adverse effects on pregnant women with GDM or HDP, including an increased risk of complications such as premature delivery and low birth weight (18). Despite the high incidence of ICP in southwest China, no research has investigated the relationship between home quarantine and ICP (27, 28). To confirm the impacts of home quarantine during COVID-19 on pregnant women with ICP, a group of pregnant women with ICP was selected and evaluated for the effects on their pregnancy outcomes as well as whether appropriate medication could improve these impacts. The findings indicated a significant increase in the incidence of ICP among pregnant women during home quarantine. While further studies are needed to confirm this result, the implications should draw attention from pregnant women, their families, the government, and medical institutions.

It has been reported that serum selenium levels generally decrease with the progression of pregnancy, and women with ICP typically exhibit lower selenium levels (29). Further studies have confirmed that low or marginal dietary selenium levels increase the risk of ICP (30). The dietary habits of the Chinese population underwent significant changes during the home quarantine period, with a marked decrease in the consumption of selenium-rich foods such as fresh vegetables and fruits, poultry, meat, and soy products (31, 32). Additionally, the sudden imposition of home quarantine measures had a severe impact on the mental health of vulnerable groups, particularly pregnant women, who were concerned about issues such as income, social relationships, and the health of their unborn child. These negative emotions contributed to increased levels of tension, anxiety, and depression, which may have raised the levels of estrogen and progesterone during pregnancy, thereby heightening the risk of ICP (33, 34). On the other hand, the alarming state of mental health makes pregnant women more prone to emotional eating behavior, eating more and tending to eat foods high in fat, carbohydrate, or energy. An unhealthy diet not only raises blood glucose and lipids but also inversely increases the risk of depression and anxiety (35, 36). Moreover, the reduction in exercise during home quarantine further exacerbates the accumulation of maternal blood sugar and blood lipids (37). Bile acids play a crucial role in regulating the homeostasis of postprandial carbohydrate and lipid metabolism (38, 39). The disruption of metabolic homeostasis due to maternal blood sugar and lipid levels may lead to an increase in bile acid levels, resulting in a higher risk of ICP (40–42). Although multiple factors associated with home quarantine could contribute to the rising incidence of ICP, more evidence is required to establish causality.

Pregnant women with ICP in the home quarantine group tended to gain more weight, which could be closely related to the changes in diet and exercise habits. Additionally, they were less likely to develop significant skin itch symptoms, which may make it harder for them to recognize their illness early on. Analysis of pregnancy outcomes indicated that the home quarantine group had a higher prevalence of GDM A1 than GDM A2, and there was also a tendency for pre-eclampsia incidence to increase. Previous studies suggest that this could be due to the higher incidence of ICP (19, 43, 44). Furthermore, we noted a higher risk of placenta accreta spectrum and postpartum bleeding among women in the home quarantine group. Several studies indicate that women with ICP have a higher likelihood of developing postpartum hemorrhage, and the placenta accreta spectrum is also an important risk factor for postpartum hemorrhage (45, 46).

The analysis of neonatal outcomes revealed that home quarantine was associated with an increased incidence of low birth weight, FGR, preterm delivery, and even stillbirth. These findings confirm previous observations made by the UK and Denmark during the COVID-19 quarantine but are inconsistent with reports from the US, Sweden, and Ireland (47–51). We also found a higher incidence of meconium-stained amniotic fluid in the home quarantine group, which may be related to obstetric factors (e.g., higher probability of HDP incidence during home quarantine) or medical factors (e.g., colonic motility due to high bile acid levels in women with ICP during pregnancy) (8). Severe meconium-stained amniotic fluid has been identified to increase the risk of various obstetric complications, such as GDM, postpartum hemorrhage, amniotic fluid excess, and intensive care unit admission, which was consistent with the adverse pregnancy outcomes we observed (52, 53). Meconium-stained amniotic fluid is often accompanied by a higher probability of polyhydramnios as well as the presence of the umbilical cord around the neck or umbilical cord knot (54). The excess amniotic fluid increases the available space for the fetus to move within the uterus and compresses the umbilical cord, ultimately increasing the risk of the cord wrapping around the neck.

Currently, a variety of drugs are used in the clinical treatment of ICP; UDCA is the first-line drug, while polyene phosphatidylcholine and S-adenosyl methionine are often used as second-line drug adjuvant therapy. However, studies on improving pregnancy outcomes and clinical symptoms have produced limited and contradictory results. Therefore, we further investigated the effects of drug use on the adverse pregnancy outcome in the home quarantine group, by using the PSM to balance the baseline differences between the medication and the non-medication groups. The results showed that the medication group had a lower risk of pre-eclampsia, polyhydramnios, meconium-stained amniotic fluid, intensive care unit admission, and umbilical cord around the neck compared to the non-medication group. It is worth noting that high bile acid is a significant risk factor for pre-eclampsia (14). The incidence of pre-eclampsia decreases when treated with hypoechoic acid in the medication group, consistent with the results of an existing randomized controlled trial (RCT) on the treatment of UDCA (55). In addition, a small RCT study showed that UDCA treatment reduced the premature delivery rate and meconium-stained amniotic fluid in fetuses of women with ICP, which has also been further confirmed in this study (56, 57). The reduced incidence of umbilical cord around the neck or umbilical cord knots in the medication group may be related to the lower incidence of polyhydramnios. Our results show that pregnant women treated with drugs usually had higher bile acid and an intense itch. However, they showed low bile acid levels during delivery, which may have been a result of drug treatment. Therefore, our findings suggest that rational drug use in women with ICP may reduce the risk of premature delivery, meconium-stained amniotic fluid, and umbilical cord around the neck (or umbilical cord knot), which could ultimately decrease the likelihood of neonatal intensive care unit admission.

In conclusion, our results suggested that home quarantine exacerbated the condition of already vulnerable women with ICP. The women with ICP in the home quarantine group had higher bile acid levels and more adverse pregnancy outcomes as compared to the control group, such as GDM A1, pre-eclampsia, postpartum hemorrhage, placenta accreta spectrum, and polyhydramnios. Their fetus was more prone to low birth weight, FGR, meconium-stained amniotic fluid, premature birth, neonatal intensive care unit admission, and even stillbirth. Preeclampsia, preterm delivery, fetal distress, meconium-stained amniotic fluid, and admission of neonates to intensive care units may be reduced through the rational use of ICP drugs. Therefore, pregnant women with ICP during home quarantine need more medical and health care. Hospitals should enhance their prenatal guidance and the management of pregnant women during public health emergencies.

### Limitations

Although this is the first large-scale cohort study about the impacts of home quarantine on pregnancy outcomes in pregnant women with ICP, there are still some limitations: (i) We failed to collect the psychological changes of pregnant women with ICP during the lockdown, and the missing medical treatment or doctor visits due to quarantine policy may reduce the detection rate of ICP. (ii) This is a retrospective cohort study, so it is difficult to obtain and quantify the diet, exercise, and mental state of pregnant women with ICP, limiting our further understanding of how home quarantine affects pregnancy outcomes. (iii) Although we use PSM and other methods to imitate the actual situation, there is still a gap in RCT research. Thus, more evidence is needed to evaluate the therapeutic effects of the drugs.

## Data availability statement

The raw data supporting the conclusions of this article will be made available by the authors, without undue reservation.

## Ethics statement

The studies involving human participants were reviewed and approved by Chongqing Medical University (ID: 20220627). The patients/participants provided their written informed consent to participate in this study.

## Author contributions

Q-YC, XLa, and T-HL: conceptualization. Q-YC, XLi, YY, and T-HL: methodology. Q-YC, YY, and XLu: software. XLi, YY, and S-JL: validation. XLi, Z-YH, YC, Y-WM, J-YH, and SY: investigation. XLu, JX, XLa, and T-HL: resources. Q-YC, XLi, and T-HL: data curation and writing—original draft preparation. XLa and T-HL: writing—review and editing and project administration. Q-YC, XLi, and S-JL: visualization. JX, XLa, and T-HL: supervision. XLu and T-HL: funding acquisition. All authors have read and agreed to the published version of the manuscript.

## References

[1] Mehandru S Merad M. Pathological sequelae of long-haul COVID. Nat Immunol. (2022) 23:194–202. 10.1038/s41590-021-01104-y 35105985 PMC9127978

[2] Rossi R Socci V Talevi D Mensi S Niolu C Pacitti F. COVID-19 pandemic and lockdown measures impact on mental health among the general population in Italy. Front Psychiatry. (2020) 11:790. 10.3389/fpsyt.2020.00790 32848952 PMC7426501

[3] Leone MJ Sigman M Golombek DA. Effects of lockdown on human sleep and chronotype during the COVID-19 pandemic. Curr Biol. (2020) 30:R930–R1. 10.1016/j.cub.2020.07.015 32810450 PMC7342078

[4] Yang X Song B Wu A Mo PKH Di J Wang Q. Social, cognitive, and ehealth mechanisms of COVID-19-related lockdown and mandatory quarantine that potentially affect the mental health of pregnant women in China: cross-sectional survey study. J Med Internet Res. (2021) 23:e24495. 10.2196/24495 33302251 PMC7836909

[5] Cai QY Yang Y Wang YH Cui HL Wu XP Liao KM. Home quarantine: a double-edged sword during COVID-19 pandemic for hypertensive disorders of pregnancy and the related complications. Diabetes Metab Syndr Obes. (2022) 15:2405–15. 10.2147/DMSO.S374482 35971524 PMC9375559

[6] Cai QY Yang Y Ruan LL Wang DD Cui HL Yang S. Effects of COVID-19 home quarantine on pregnancy outcomes of patients with gestational diabetes mellitus: a retrospective cohort study. J Matern Fetal Neonatal Med. (2023) 36:2193284. 10.1080/14767058.2023.2193284 36977601

[7] Ovadia C Seed PT Sklavounos A Geenes V Di Ilio C Chambers J. Association of adverse perinatal outcomes of intrahepatic cholestasis of pregnancy with biochemical markers: results of aggregate and individual patient data meta-analyses. Lancet. (2019) 393:899–909. 10.1016/S0140-6736(18)31877-4 30773280 PMC6396441

[8] From From the American Association of Neurological Surgeons ASoNC Interventional Interventional Radiology Society of Europe CIRACoNSESoMINTESoNESOSfCA Interventions SoIRSoNS World Stroke O Sacks D Baxter B . Multisociety consensus quality improvement revised consensus statement for endovascular therapy of acute ischemic stroke. Int J Stroke. (2018) 13:612–32. 10.1177/1747493018778713 29786478

[9] Terrault NA Williamson C. Pregnancy-associated liver diseases. Gastroenterology. (2022) 163:97–117 e1. 10.1053/j.gastro.2022.01.060 35276220

[10] Davenport MH Ruchat SM Poitras VJ Jaramillo Garcia A Gray CE Barrowman N. Prenatal exercise for the prevention of gestational diabetes mellitus and hypertensive disorders of pregnancy: a systematic review and meta-analysis. Br J Sports Med. (2018) 52:1367–75. 10.1136/bjsports-2018-099355 30337463

[11] Manzotti C Casazza G Stimac T Nikolova D Gluud C. Total serum bile acids or serum bile acid profile, or both, for the diagnosis of intrahepatic cholestasis of pregnancy. Cochrane Database Syst Rev. (2019) 7:CD012546. 10.1002/14651858.CD012546.pub2 31283001 PMC6613619

[12] Bicocca MJ Sperling JD Chauhan SP. Intrahepatic cholestasis of pregnancy: Review of six national and regional guidelines. Eur J Obstet Gynecol Reprod Biol. (2018) 231:180–7. 10.1016/j.ejogrb.2018.10.041 30396107

[13] Wu K Yin B Li S Zhu X Zhu B. Prevalence, risk factors and adverse perinatal outcomes for Chinese women with intrahepatic cholestasis of pregnancy: a large cross-sectional retrospective study. Ann Med. (2022) 54:2966–74. 10.1080/07853890.2022.2136400 36271887 PMC9624205

[14] Deng W Zhang L Du Q Li Y Chen J Du L. The association of serum total bile acid with new-onset hypertension during pregnancy. BMC Pregnancy Childbirth. (2022) 22:879. 10.1186/s12884-022-05211-y 36435758 PMC9701419

[15] Chmielewska B Barratt I Townsend R Kalafat E van der Meulen J Gurol-Urganci I. Effects of the COVID-19 pandemic on maternal and perinatal outcomes: a systematic review and meta-analysis. Lancet Glob Health. (2021) 9:e759–e72. 10.1016/S2214-109X(21)00079-6 33811827 PMC8012052

[16] Ceulemans M Foulon V Ngo E Panchaud A Winterfeld U Pomar L. Mental health status of pregnant and breastfeeding women during the COVID-19 pandemic-a multinational cross-sectional study. Acta Obstet Gynecol Scand. (2021) 100:1219–29. 10.1111/aogs.14092 33475148 PMC8014496

[17] Goyal LD Garg P Verma M Kaur N Bakshi D Arora J. Effect of restrictions imposed due to COVID-19 pandemic on the antenatal care and pregnancy outcomes: a prospective observational study from rural North India. BMJ Open. (2022) 12:e059701. 10.1136/bmjopen-2021-059701 35387835 PMC8987212

[18] Liu Y Dai M Tang S. Effect of initial COVID-19 outbreak during first trimester on pregnancy outcome in Wuxi, China. BMC Pregnancy Childbirth. (2022) 22:54. 10.1186/s12884-022-04395-7 35062910 PMC8778492

[19] Aghili R Honardoost M Khamseh ME. COVID-19: Case fatality and ACE2 inhibitors treatment concerns in patients with comorbidities. Med J Islam Repub Iran. (2020) 34:147. 10.47176/mjiri.34.147 33437743 PMC7787016

[20] Hughes DS Magann EF Whittington JR Wendel MP Sandlin AT Ounpraseuth ST. Accuracy of the ultrasound estimate of the amniotic fluid volume (amniotic fluid index and single deepest pocket) to identify actual low, normal, and high amniotic fluid volumes as determined by quantile regression. J Ultrasound Med. (2020) 39:373–8. 10.1002/jum.15116 31423632

[21] Wax JR Pinette MG. The amniotic fluid index and oligohydramnios: a deeper dive into the shallow end. Am J Obstet Gynecol. (2022) 227:462–70. 10.1016/j.ajog.2022.04.016 35452652

[22] International International Association of D Pregnancy Study Groups Consensus P Metzger BE Gabbe SG Persson B Buchanan TA. International association of diabetes and pregnancy study groups recommendations on the diagnosis and classification of hyperglycemia in pregnancy. Diabetes Care. (2010) 33:676–82. 10.2337/dc09-1848 20190296 PMC2827530

[23] Brown MA Magee LA Kenny LC Karumanchi SA McCarthy FP Saito S. The hypertensive disorders of pregnancy: ISSHP classification, diagnosis and management recommendations for international practice. Pregnancy Hypertens. (2018) 13:291–310. 10.1161/HYPERTENSIONAHA.117.10803 29803330

[24] Loescher T Allum J Phillips N. Prudent healthcare in practice: integration of audiology services into primary care. BMJ Open Qual. (2022) 11:3. 10.1136/bmjoq-2022-001884 36008045 PMC9422886

[25] Society for Maternal-Fetal Medicine Electronic Address Pso Lee RH Mara G Metz TD Pettker CM. Society for maternal-fetal medicine consult series #53: intrahepatic cholestasis of pregnancy: replaces consult #13, April 2011. Am J Obstet Gynecol. (2021) 224:B2–B9. 10.1016/j.ajog.2020.11.002 33197417

[26] Nguyen TL Collins GS Spence J Daures JP Devereaux PJ Landais P. Double-adjustment in propensity score matching analysis: choosing a threshold for considering residual imbalance. BMC Med Res Methodol. (2017) 17:78. 10.1186/s12874-017-0338-0 28454568 PMC5408373

[27] Mikolasevic I Filipec-Kanizaj T Jakopcic I Majurec I Brncic-Fischer A Sobocan N. Liver disease during pregnancy: a challenging clinical issue. Med Sci Monit. (2018) 24:4080–90. 10.12659/MSM.907723 29905165 PMC6034557

[28] Smith DD Rood KM. Intrahepatic cholestasis of pregnancy. Clin Obstet Gynecol. (2020) 63:134–51. 10.1097/GRF.0000000000000495 31764000

[29] Reyes H Baez ME Gonzalez MC Hernandez I Palma J Ribalta J. Selenium, zinc and copper plasma levels in intrahepatic cholestasis of pregnancy, in normal pregnancies and in healthy individuals, in Chile. J Hepatol. (2000) 32:542–9. 10.1016/S0168-8278(00)80214-7 10782901

[30] Floreani A Gervasi MT. New insights on intrahepatic cholestasis of pregnancy. Clin Liver Dis. (2016) 20:177–89. 10.1016/j.cld.2015.08.010 26593298

[31] Clemente-Suarez VJ Ramos-Campo DJ Mielgo-Ayuso J Dalamitros AA Nikolaidis PA Hormeno-Holgado A. Nutrition in the actual COVID-19 pandemic. a narrative review. Nutrients. (2021) 13:6. 10.3390/nu13061924 34205138 PMC8228835

[32] Jia P Liu L Xie X Yuan C Chen H Guo B. Changes in dietary patterns among youths in China during COVID-19 epidemic: the COVID-19 impact on lifestyle change survey (COINLICS). Appetite. (2021) 158:105015. 10.1016/j.appet.2020.105015 33121998

[33] Borges Manna L Williamson C. Nuclear receptors in pregnancy and outcomes: clinical perspective. Adv Exp Med Biol. (2022) 1390:3–19. 10.1007/978-3-031-11836-4_1 36107310

[34] Ebrahim S Ashworth H Noah C Kadambi A Toumi A Chhatwal J. Reduction of COVID-19 incidence and nonpharmacologic interventions: analysis using a us county-level policy data set. J Med Internet Res. (2020) 22:e24614. 10.2196/24614 33302253 PMC7755429

[35] Firth J Gangwisch JE Borisini A Wootton RE Mayer EA. Food and mood: how do diet and nutrition affect mental wellbeing? BMJ. (2020) 369:m2382. 10.1136/bmj.m2382 32601102 PMC7322666

[36] Mattioli AV Sciomer S Cocchi C Maffei S Gallina S. Quarantine during COVID-19 outbreak: changes in diet and physical activity increase the risk of cardiovascular disease. Nutr Metab Cardiovasc Dis. (2020) 30:1409–17. 10.1016/j.numecd.2020.05.020 32571612 PMC7260516

[37] Niela-Vilen H Auxier J Ekholm E Sarhaddi F Asgari Mehrabadi M Mahmoudzadeh A. Pregnant women's daily patterns of well-being before and during the COVID-19 pandemic in Finland: Longitudinal monitoring through smartwatch technology. PLoS ONE. (2021) 16:e0246494. 10.1371/journal.pone.0246494 33534854 PMC7857616

[38] Clifford BL Sedgeman LR Williams KJ Morand P Cheng A Jarrett KE. FXR activation protects against NAFLD via bile-acid-dependent reductions in lipid absorption. Cell Metab. (2021) 33:1671–84 e4. 10.1016/j.cmet.2021.06.012 34270928 PMC8353952

[39] de Vos WM Tilg H Van Hul M Cani PD. Gut microbiome and health: mechanistic insights. Gut. (2022) 71:1020–32. 10.1136/gutjnl-2021-326789 35105664 PMC8995832

[40] Song F Chen Y Chen L Li H Cheng X Wu W. Association of elevated maternal serum total bile acids with low birth weight and intrauterine fetal growth restriction. JAMA Netw Open. (2021) 4:e2117409. 10.1001/jamanetworkopen.2021.17409 34279647 PMC8290304

[41] Zhan Y Xu T Chen T Wang X. Intrahepatic cholestasis of pregnancy and maternal dyslipidemia: a systematic review and meta-analysis. Acta Obstet Gynecol Scand. (2022) 101:719–27. 10.1111/aogs.14380 35599353 PMC9564710

[42] Zhang Y Lan X Cai C Li R Gao Y Yang L. Associations between maternal lipid profiles and pregnancy complications: a prospective population-based study. Am J Perinatol. (2021) 38:834–40. 10.1055/s-0039-3402724 31891957

[43] Liu C Gao J Liu J Wang X He J Sun J. Intrahepatic cholestasis of pregnancy is associated with an increased risk of gestational diabetes and preeclampsia. Ann Transl Med. (2020) 8:1574. 10.21037/atm-20-4879 33437773 PMC7791254

[44] de la Torre NG Assaf-Balut C Jimenez Varas I Del Valle L Duran A Fuentes M. Effectiveness of following mediterranean diet recommendations in the real world in the incidence of gestational diabetes mellitus (GDM) and adverse maternal-foetal outcomes: a prospective, universal, interventional study with a single group. The St Carlos study. Nutrients. (2019) 11:6. 10.3390/nu11061210 31141972 PMC6627921

[45] Griffiths LA Flatters SJ. Pharmacological modulation of the mitochondrial electron transport chain in paclitaxel-induced painful peripheral neuropathy. J Pain. (2015) 16:981–94. 10.1016/j.jpain.2015.06.008 26142652 PMC4596251

[46] Arthuis C Diguisto C Lorphelin H Dochez V Simon E Perrotin F. Perinatal outcomes of intrahepatic cholestasis during pregnancy: an 8-year case-control study. PLoS ONE. (2020) 15:e0228213. 10.1371/journal.pone.0228213 32074108 PMC7029845

[47] Pasternak B Neovius M Soderling J Ahlberg M Norman M Ludvigsson JF. Preterm birth and stillbirth during the COVID-19 pandemic in Sweden: a nationwide cohort study. Ann Intern Med. (2021) 174:873–5. 10.7326/M20-6367 33428442 PMC7808327

[48] Hedermann G Hedley PL Baekvad-Hansen M Hjalgrim H Rostgaard K Poorisrisak P. Danish premature birth rates during the COVID-19 lockdown. Arch Dis Child Fetal Neonatal. (2021) 106:93–5. 10.1136/archdischild-2020-319990 32788391 PMC7421710

[49] Khalil A von Dadelszen P Draycott T Ugwumadu A O'Brien P Magee L. Change in the incidence of stillbirth and preterm delivery during the COVID-19 pandemic. JAMA. (2020). 10.1001/jama.2020.12746 32648892 PMC7435343

[50] Philip RK Purtill H Reidy E Daly M Imcha M McGrath D. Unprecedented reduction in births of very low birthweight (VLBW) and extremely low birthweight (ELBW) infants during the COVID-19 lockdown in Ireland: a 'natural experiment' allowing analysis of data from the prior two decades. BMJ Glob Health. (2020) 5:e003075. 10.1136/bmjgh-2020-003075 32999054 PMC7528371

[51] Handley SC Mullin AM Elovitz MA Gerson KD Montoya-Williams D Lorch SA. Changes in preterm birth phenotypes and stillbirth at 2 Philadelphia hospitals during the SARS-CoV-2 pandemic, March–June 2020. JAMA. (2021) 325:87–9. 10.1001/jama.2020.20991 33284323 PMC7786240

[52] Fang ZJ Liu HF Zhang YL Yu L Yan JY. Relation of meconium-stained amniotic fluid and postpartum hemorrhage: a retrospective cohort study. Eur Rev Med Pharmacol Sci. (2020) 24:10352–8. 10.26355/eurrev_202010_23384 33155191

[53] Levin G Tsur A Shai D Cahan T Shapira M Meyer R. Prediction of adverse neonatal outcome among newborns born through meconium-stained amniotic fluid. Int J Gynaecol Obstet. (2021) 154:515–20. 10.1002/ijgo.13592 33448026

[54] Chiou YL Hung CH Liao HY. The impact of prepregnancy body mass index and gestational weight gain on perinatal outcomes for women with gestational diabetes mellitus. Worldviews Evid Based Nurs. (2018) 15:313–22. 10.1111/wvn.12305 29962105

[55] Chappell LC Chambers J Dixon PH Dorling J Hunter R Bell JL. Ursodeoxycholic acid versus placebo in the treatment of women with intrahepatic cholestasis of pregnancy (ICP) to improve perinatal outcomes: protocol for a randomised controlled trial (PITCHES). Trials. (2018) 19:657. 10.1186/s13063-018-3018-4 30482254 PMC6260710

[56] Chappell LC Bell JL Smith A Linsell L Juszczak E Dixon PH. Ursodeoxycholic acid vs. placebo in women with intrahepatic cholestasis of pregnancy (PITCHES): a randomised controlled trial. Lancet. (2019) 394:849–60. 10.1016/S0140-6736(19)31270-X 31378395 PMC6739598

[57] Bacq Y Sentilhes L Reyes HB Glantz A Kondrackiene J Binder T. Efficacy of ursodeoxycholic acid in treating intrahepatic cholestasis of pregnancy: a meta-analysis. Gastroenterology. (2012) 143:1492–501. 10.1053/j.gastro.2012.08.004 22892336

