# Supplementary material for: Rational use of drugs to alleviate adverse outcomes caused by COVID-19 quarantine in women with intrahepatic cholestasis of pregnancy
Source: Front Med (Lausanne). 2023 Aug 7;10:1122873. doi: 10.3389/fmed.2023.1122873 (PMC10441112; doi:10.3389/fmed.2023.1122873)
Supplement: Supplementary file 1 [file Data_Sheet_1.docx]

**
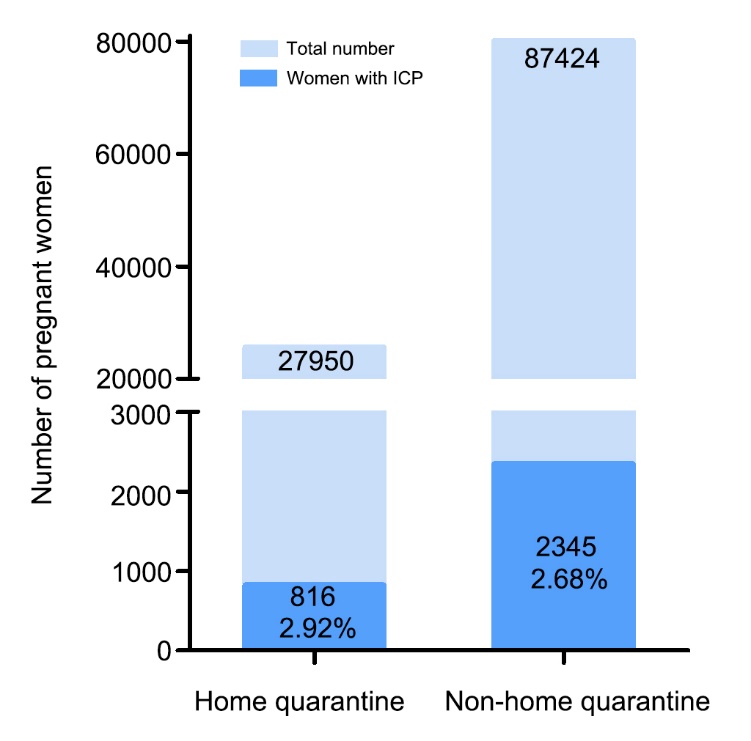
**

**Figure S1. The total delivery and women with ICP in the home quarantine group and the non- home quarantine group in 2018, 2019, 2020, and 2021.**

**Table S1. Sociodemographic and obstetric histories of patients with ICP in the home quarantine and non-home quarantine groups.**

| **Characteristics** | **Home quarantine** | **Non-home quarantine** | ***X^2^/F*** | ***P*** |
| --- | --- | --- | --- | --- |
| **Total** | 816 | 2345 |  |  |
| **Maternal age (years)** | 29.97±0.15 | 30.21±0.10 | 1.45 | *P*=0.229 |
| **Gestational weight gain** † | 16.50(14.00-20.00) | 16.00(13.00-19.00) | 4.44 | *P*=0.035* |
| **Gestational age** | 38.00(37.00-38.32) | 37.76(37.00-38.43) | 1.29 | *P*=0.256 |
| **Gravidity** |  |  | 0.37 | *P*=0.545 |
| 1 | 323(39.6%) | 937(39.9%) |  |  |
| 2 | 237(29.0%) | 639(27.3%) |  |  |
| 3 | 138(16.9%) | 377(16.1%) |  |  |
| ≥4 | 118(14.5%) | 392(16.7%) |  |  |
| **Parity** |  |  | 0.01 | *P*=0.979 |
| 0 | 563(69.0%) | 1625(69.3%) |  |  |
| 1 | 225(27.6%) | 637(27.2%) |  |  |
| ≥2 | 28(3.4%) | 83(3.5%) |  |  |
| **Abortion** |  |  | 0.05 | *P*=0.826 |
| 0 | 503(61.6%) | 1425(60.8%) |  |  |
| 1 | 167(20.5%) | 504(21.5%) |  |  |
| ≥2 | 146(17.7%) | 416(17.7%) |  |  |
| **Pre-gestation BMI (kg/m^2^)** |  |  | 2.61 | *P*=0.106 |
| <18.5 | 149(18.3%) | 412(17.6%) |  |  |
| 18.5-23.9 | 569(69.7%) | 1569(66.9%) |  |  |
| 24-27.9 | 84(10.3%) | 306(13.0%) |  |  |
| >28 | 14(1.7%) | 58(2.5%) |  |  |
| **Birth plurality** † |  |  | 4.56 | *P*=0.033* |
| Singletons | 659(80.8%) | 1970(84.0%) |  |  |
| Twins | 157(19.2%) | 375(16.0%) |  |  |
|  |  |  |  |  |
| **Cigarette smoking** |  |  | 1.97 | *P*=0.160 |
| Yes | 4(0.5%) | 24(1.0%) |  |  |
| No | 812(99.5%) | 2321(99.0%) |  |  |
| **Alcohol consumption** |  |  | 0.98 | *P*=0.323 |
| Yes | 2(0.2%) | 12(0.5%) |  |  |
| No | 814(99.8%) | 2333(99.5%) |  |  |
| **Severity of intrahepatic cholestasis of pregnancy** |  |  | 1.52 | *P*=0.218 |
| Mild | 500(61.3%) | 1381(58.9%) |  |  |
| Severe | 316(38.7%) | 964(41.4%) |  |  |
| **History of hepatobiliary disease** |  |  | 1.67 | *P*=0.196 |
| Yes | 55(6.7%) | 124(5.3%) |  |  |
| No | 761(93.3%) | 2221(94.7%) |  |  |
| **History of intrahepatic cholestasis of pregnancy** |  |  | 0.06 | *P*=0.811 |
| Yes | 20(3.7%) | 82(3.5%) |  |  |
| No | 786(96.3%) | 2263(96.5%) |  |  |
| **History of abnormal gestation and birth** |  |  | 0.69 | *P*=0.405 |
| Yes | 48(5.9%) | 156(6.7%) |  |  |
| No | 768(94.1%) | 2189(93.3%) |  |  |
| **Assisted reproductive technology** |  |  | 0.18 | *P*=0.674 |
| Yes | 109(13.4%) | 300(12.8%) |  |  |
| No | 707(86.6%) | 2045(87.2%) |  |  |
| **Using of medication** † |  |  | 15.95 | *P*<0.001*** |
| Yes | 322(39.5%) | 1116(47.6%) |  |  |
| No | 493(60.5%) | 1229(52.4%) |  |  |
| **Skin itch** † |  |  | 16.69 | *P<*0.001*** |
| Yes | 252(30.9%) | 912(38.9%) |  |  |
| No | 564(69.1%) | 1433(61.1%) |  |  |

**Home quarantine was used as reference group.**

**†: There was statistically significant difference between the home quarantine group and the non-home quarantine group.**

***: *P*<0.05, **: *P*<0.01, ***: *P*<0.001**

**Table S2.** **Maternal serum biochemistry of patients with ICP** **in the home quarantine and non-home quarantine groups.**

|  | **Level at time of diagnosis** | ***P*** | **Gestation age at measurement** | **Level at time of** **delivery** | ***P*** | **Gestation age at measurement** | **reference range for pregnancy^*^** |
| --- | --- | --- | --- | --- | --- | --- | --- |
| **Serum bile acids (μ mol/L)** |  | *P*=0.049* |  |  | *P*=0.921 |  | 0-10μmol/L |
| Home quarantine | 27.4(17.1-62.1) |  | 35.43(32.43-37.86) | 23.2(12.3-58.6) |  | 38.00(37.00-38.32) |  |
| Non-home quarantine | 24.9(14.7-63.6) |  | 35.00(32.43-37.21) | 22.6(12.1-55.2) |  | 37.76(37.00-38.43) |  |
| **Alanine transaminase (IU/L)** |  | *P*=0.741 |  |  | *P*=0.061 |  | 7-40 IU/L |
| Home quarantine | 162(83.0-380.4) |  | 35.43(32.43-37.86) | 130.0(52.0-357.2) |  | 38.00(37.00-38.32) |  |
| Non-home quarantine | 156.3(80.5-365.2) |  | 35.00(32.43-37.21) | 122.0(35.0-349.1) |  | 37.76(37.00-38.43) |  |
| **Aspartate transaminase (IU/L)** |  | *P*=0.550 |  |  | *P*=0.062 |  | 13-35 IU/L |
| Home quarantine | 105.0(58-174.0) |  | 35.43(32.43-37.86) | 90.8(41.5-237.1) |  | 38.00(37.00-38.32) |  |
| Non-home quarantine | 104.0(57.0-171.9) |  | 35.00(32.43-37.21) | 81.0(36.0-216.2) |  | 37.76(37.00-38.43) |  |
| **Total Bilirubin (μ mol/L)** |  | *P*=0.518 |  |  | *P*=0.772 |  | 5.1-28μmol/L |
| Home quarantine | 15.1(12.4-26.2) |  | 35.43(32.43-37.86) | 11.6(8.3-20.4) |  | 38.00(37.00-38.32) |  |
| Non-home quarantine | 14.4(10.3-21.6) |  | 35.00(32.43-37.21) | 11.2(7.8-20.3) |  | 37.76(37.00-38.43) |  |

**All results were given as median (IQR).**

**^*^Please note that some hospitals use different reference ranges.**

***: *P*<0.05.**

**Table S3. Sociodemographic and obstetric histories of patients with ICP who used medication or without medication in the home quarantine group.**

| **Characteristics** | **Medication** | **Non-medication** | ***X^2^/F*** | ***P*** |
| --- | --- | --- | --- | --- |
| **Total** | 311 | 311 |  |  |
| **Maternal age (years)** | 29.80±021 | 30.02±0.22 | 0.01 | *P*=0.976 |
| **Gestational weight gain** | 12.79±0.33 | 13.68±0.32 | 0.25 | *P*=0.615 |
| **Gestational age** | 38.86(37.86-39.71) | 38.46(37.64-39.43) | 0.38 | *P*=0.538 |
| **Gravidity** |  |  | 2.52 | *P*=0.112 |
| 1 | 115(37.0%) | 133(42.8%) |  |  |
| 2 | 87(28.0%) | 83(26.7%) |  |  |
| 3 | 59(19.0%) | 51(16.4%) |  |  |
| ≥4 | 50(16.0%) | 44(14.1%) |  |  |
| **Parity** |  |  | 3.80 | *P*=0.149 |
| 0 | 222(71.4%) | 243(78.1%) |  |  |
| 1 | 77(24.8%) | 58(18.6%) |  |  |
| ≥2 | 12(3.9%) | 10(3.2%) |  |  |
| **Abortion** † |  |  | 1.69 | *P*=0.194 |
| 0 | 184(59.2%) | 200(64.3%) |  |  |
| 1 | 71(22.8%) | 61(19.6%) |  |  |
| ≥2 | 56(18.0%) | 50(16.1%) |  |  |
| **Pre-gestation BMI (kg/m^2^)** |  |  | 0.26 | *P*=0.611 |
| <18.5 | 55(17.7%) | 50(16.1%) |  |  |
| 18.5-23.9 | 213(70.1%) | 221(71.1%) |  |  |
| 24-27.9 | 32(10.3%) | 35(11.3%) |  |  |
| >28 | 6(1.9%) | 5(1.6%) |  |  |
| **Birth plurality** |  |  | 1.45 | *P*=0.270 |
| Singletons | 243(78.1%) | 255(82.0%) |  |  |
| Twins | 68(21.9%) | 56(18.0%) |  |  |
| **Cigarette smoking** |  |  |  |  |
| Yes | 1(0.3%) | 3(1.0%) | 1.01 | *P*=0.316 |
| No | 310(99.7%) | 308(99.0%) |  |  |
| **Alcohol consumption** |  |  |  |  |
| Yes | 0(0.0%) | 2(0.6%) | 2.01 | *P*=0.157 |
| No | 311(100.0%) | 309(99.4%) |  |  |
| **Severity of intrahepatic cholestasis of pregnancy** |  |  | 0.54 | *P*=0.463 |
| Mild | 188(60.5%) | 179(57.6%) |  |  |
| Severe | 123(39.5%) | 132(42.4%) |  |  |
| **History of hepatobiliary disease** |  |  | 0.02 | *P*=0.879 |
| Yes | 23(7.4%) | 24(7.7%) |  |  |
| No | 288(92.6%) | 287(92.3%) |  |  |
| **History of intrahepatic cholestasis of pregnancy** |  |  | 2.66 | *P*=0.103 |
| Yes | 13(4.2%) | 6(1.9%) |  |  |
| No | 298(95.8%) | 305(98.1%) |  |  |
| **History of abnormal gestation and birth** |  |  | 2.67 | *P*=0.102 |
| Yes | 15(4.8%) | 25(8.0%) |  |  |
| No | 296(95.2%) | 286(92.0%) |  |  |
| **Assisted reproductive technology** |  |  | 2.41 | *P*=0.120 |
| Yes | 41(13.2%) | 55(17.7%) |  |  |
| No | 270(86.8%) | 256(82.3%) |  |  |
| **Skin itch** † |  |  | 11.36 | *P*=0.001** |
| Yes | 117(37.6%) | 78(25.1%) |  |  |
| No | 194(62.4%) | 233(74.9%) |  |  |

**Using of medication was used as reference group.**

**†: There was statistically significant difference between the medication group and non-medication group.**

***: *P*<0.05, **: *P*<0.01, ***: *P*<0.001**

**Table S4. Maternal serum biochemistry of patients with ICP with or without medication in the home quarantine group.**

|  | **Level at time of diagnosis** | ***P*** | **Gestation age at measurement** | **Level at time of delivery** | ***P*** | **Gestation age at measurement** | **reference range for pregnancy^*^** |
| --- | --- | --- | --- | --- | --- | --- | --- |
| **Serum bile acids (μ mol/L)** |  | *P*=0.271 |  |  | *P*=0.042* |  | 0-10μmol/L |
| Medication | 26.2(15.7-63.4) |  | **35.14(33.57-38.0)** | 20.4(12.4-49.6) |  | **38.86(37.86,39.71)** |  |
| Non-medication | 24.6(13.7-65.2) |  | **35.86(34.86-38.35)** | 22.6(15.5-53.5) |  | **38.86(37.86,39.43)** |  |
| **Alanine transaminase (IU/L)** |  | *P*=0.212 |  |  | *P*=0.138 |  | 7-40 IU/L |
| Home quarantine | 165.5(83-392.5) |  | **35.14(33.57-38.0)** | 100.2(38.0-289.2) |  | **38.86(37.86,39.71)** |  |
| Non-home quarantine | 141(79.0-338.8) |  | **35.86(34.86-38.35)** | 127.0(49.0-423.0) |  | **38.86(37.86,39.43)** |  |
| **Aspartate transaminase (IU/L)** |  | *P*=0.218 |  |  | *P*=0.025* |  | 13-35 IU/L |
| Home quarantine | 110.0(58.5-231.8) |  | **35.14(33.57-38.0)** | 69.4(34.0-157.8) |  | **38.86(37.86,39.71)** |  |
| Non-home quarantine | 101(55.0-199.4) |  | **35.86(34.86-38.35)** | 84.0(42.0-250.4) |  | **38.86(37.86,39.43)** |  |
| **Total Bilirubin (μ mol/L)** |  | *P*=0.850 |  |  | *P*=0.003** |  | 5.1-28μmol/L |
| Home quarantine | 10.8(5.3-21.6 |  | **35.14(33.57-38.0)** | 10.9(7.7-17.4) |  | **38.86(37.86,39.71)** |  |
| Non-home quarantine | 8.5(4.4-18.4) |  | **35.86(34.86-38.35)** | 11.1(9.0-22.5) |  | **38.86(37.86,39.43)** |  |

**All results were given as median (IQR).**

**^*^Please note that some hospitals use different reference ranges.**

***: *P*<0.05.**
